# Modified silica-based double-layered hydrophobic-coated stainless steel mesh and its application for oil/seawater separation

**DOI:** 10.1038/s41598-024-51264-8

**Published:** 2024-01-06

**Authors:** Aunchalee Deachophon, Thiti Bovornratanaraks, Sirilux Poompradub

**Affiliations:** 1 Department of Chemical Technology, Faculty of Science, Chulalongkorn University, Phatumwan, Bangkok, 10330 Thailand; 2 Department of Physics, Faculty of Science, Chulalongkorn University, Phatumwan, Bangkok, 10330 Thailand; 3 Center of Excellent in Green Materials for Industrial Application, Faculty of Science, Chulalongkorn University, Phatumwan, Bangkok, 10330 Thailand; 4 Center of Excellence on Petrochemical and Materials Technology, Chulalongkorn University, Pathumwan, Bangkok, 10330 Thailand

**Keywords:** Environmental sciences, Materials science

## Abstract

A double-layered hydrophobic-coated stainless steel mesh (CSSM) was successfully prepared by vapor deposition of polydimethylsiloxane (PDMS) to form aerosol silica (SiO_2_) particles on SSM followed by coating with the in situ modified SiO_2_ generated in the natural rubber (NR) latex for use in oil/seawater separation. The in situ SiO_2_ particles were modified with octyltriethoxysilane (OTES) or hexadecyltrimethoxysilane (HDTMS). Transmission electron microscopy, ^29^Si solid-state nuclear magnetic resonance, and Fourier transform infrared spectroscopy were used to determine the structure of the in situ modified SiO_2_ generated in the NR latex. Scanning electron microscopy and water contact angle analyses were applied to characterize the morphology and hydrophobicity of the CSSM, respectively. The presence of aerosol SiO_2_ particles from PDMS and in situ modified SiO_2_ by OTES (MSi-O) or HDTMS (MSi-H) generated in the NR could enhance the surface roughness and hydrophobicity of the CSSM. The hydrophobic CSSM was then applied for the separation of chloroform/seawater and crude oil/seawater mixtures. A high separation efficiency (up to 99.3%) with the PDMS/NR/MSi-H CSSM was obtained and the mesh was reusable for up to 20 cycles.

## Introduction

Accidental oil spills cause severe environmental and health issues as oil-polluted water^1^. There have been 1854 oil spills worldwide since 1970, totaling roughly 5.87 million tonnes of oil^2^. These not only result in significant economic losses but also have a negative impact on the ecosystem health, environmental pollution, and human health^3,4^. Additionally, it might harm the entire population in the long term. Thus, the effective separation of oil from water has attracted interest on a global scale. This major issue can be resolved using a variety of techniques, including burning^5,6^, skimming^7^, and the use of dispersants^8,9^. However, the above-mentioned methods require high energy consumption. Furthermore, burning generates a large amount of black smoke, which raises concerns about the effects of the smoke plume on humans, wildlife, and the environment, while the use of dispersants releases hazardous break-down products into the environment that have significant negative impacts on marine life. Therefore, simple eco-friendly and cost-effective methods are required to solve the above problem.

Currently, the materials for oil/water separation can be divided into two types, i.e., filtration (filter paper, sand, and metal mesh)^10–15^ and absorption (cotton fabric, and sponge) ^16–19^. Nevertheless, absorption materials have a complicated post-processing^20^. As a result, filtration materials are more suitable for use in oil/water separation. Metallic meshes are the most extensively studied substrates for the separation of oil/water mixtures due to their high mechanical strength, good thermal properties, and recyclability^1,21^. Various types of metals/alloys have been used, such as stainless steel, copper, and brass. Although copper and brass can react with oxygen to corrode, stainless steel has an excellent corrosion resistance^22^ and low cost compared to copper and brass.

Water repellency, such as what is seen on the surface of lotus leaves, is known as hydrophobicity. The static water contact angle (WCA) of a hydrophobic surface is about 90°–150° (water-repelling). The key factors contributing to the hydrophobic behavior of materials include surface roughness and low surface energy. Generally, a hydrophobic surface can simply be fabricated by coating with low surface energy substances. In various studies, researchers have modified the surfaces of silica (SiO_2_)^23,24^, titanium dioxide^21,25^, or zirconium dioxide^26,27^ nanoparticles (NPs). Subsequently, these modified material particles were coated onto the mesh to improve its hydrophobicity. In this research, SiO_2_ was selected due to its low toxicity, biocompatibility, simplicity to synthesize, and low cost^28^. However, SiO_2_ NPs have hydrophilic properties, so it is necessary to modify their surface so that they become hydrophobic. Although fluoroalkoxysilanes have been extensively used for modifying NPs due to their low surface energy, fluorinated compounds are highly toxic, bioaccumulate, expensive, and have been found to be carcinogenic^29,30^ For this reason, two non-fluorinated compounds, octyltriethoxysilane (OTES) and hexadecyltrimethoxysilane (HDTMS), were used in this study. Since SiO_2_ NPs cannot stick to the mesh’s surface, they were combined with natural rubber (NR) latex, which has an outstanding high elasticity and high tensile strength.

The NR latex is obtained from the Para rubber tree (*Hevea brasiliensis*), with *cis*-1,4-polyisoprene as the major component that consists of hydrophobic segments. Upon harvesting, the NR latex is supplied with ammonia to stop it from coagulation. In addition, the NR latex in this study was used as a matrix to make a good dispersion of modified SiO_2_ NPs and it could be easily coated on the stainless-steel mesh (SSM). However, the incompatibility between conventional (ex situ formed) SiO_2_ and the NR matrix causes poor mechanical properties and poor dispersion due to the surface area of ex situ SiO_2_ having a higher level of silanol groups^31,32^. To solve this problem, the generation of in situ SiO_2_ in the NR matrix by a sol–gel reaction has been widely used. Accordingly, in situ modified SiO_2_ generated in the NR latex was synthesized using the sol–gel reaction in this study. Furthermore, a few studies have reported that hydrophobic materials show a poor mechanical durability due to their low adhesion between the coating and metal surface. Therefore, polydimethylsiloxane (PDMS) is commonly used as a binder to improve the adhesion strength given that PDMS is biocompatible, inexpensive, non-toxic, and has a good adhesion property on various surface materials^33,34^. It is important to note that the important key of this research is to design a hydrophobic coated SSM that can be used in a real situation if a crude oil spill occurs in seawater, and so to determine whether the designed materials can actually be used or not. In addition, previous experiments have examined the oil separation in water^1,3,10–12,14,16–18,21,23–26^ but not in seawater.

Accordingly, this research aimed to prepare a simple and inexpensive approach to fabricate a double-layered hydrophobic coated SSM (CSSM) from PDMS through the vapor deposition and in situ modified SiO_2_ generated in the NR latex. The in situ modified SiO_2_ generated in NR latex was synthesized using tetraethyl orthosilicate (TEOS) as the silica precursor and then the SiO_2_ surface was modified by OTES or HDTMS via the sol–gel reaction. The CSSMs were shown to be hydrophobic with a rougher surface compared to the uncoated SSM ones. Scanning electron microscopy (SEM) and WCA analyses were applied to characterize the CSSMs. Finally, the mechanical durability, oil/water separation efficiency, and permeate flux of the CSSMs were also investigated.

## Materials and methods

### Materials

A 304 SSM of 150 mesh with an average pore size of about 106 µm and an opening surface of 41% was used as the substrate. The NR latex with a 60% dry rubber content (DRC) was purchased from the Thai Rubber Latex Co., Thailand. The PDMS (Sylgard 184; elastomer base with curing agent in a 10:1 mass ratio) was purchased from Dow Corning, USA, while TEOS, used as the silica precursor, was from Sigma-Aldrich, China. The modifiers, OTES and HDTMS, were purchased from Sigma-Aldrich, USA and China, respectively. Chloroform was purchased from RCI Labscan, Ireland. Crude oil was supplied by the Bangchak Petroleum Public Co., Ltd. Sudan III (Sigma-Aldrich, USA) and methylene blue (QRëC, New Zealand) were used as coloring agents. All the chemicals were used as received.

### Preparation of in situ modified SiO_2_ generated in NR latex

The in situ modified SiO_2_ was prepared through a sol–gel reaction. Firstly, 40 mL TEOS was dissolved in 3 mL deionized (DI) water for 15 min. The colloidal SiO_2_ formation was obtained. Then, 0.45 mL modifier (OTES or HDTMS) was slowly added. The mixture was stirred for 1 h at an ambient temperature (30 °C). The NR latex with a 60% DRC was diluted by DI water to obtain NR latex with 15% DRC. The mixture was then added drop-wise into the NR latex (15% DRC) and the suspension was stirred under a closed system at 700 revolutions per minute (rpm) for 24 h. For the preparation of in situ unmodified SiO_2_ in NR latex, a mixture of TEOS and DI water was stirred for 1 h without adding a modifier. Also, the mixture was added drop-wise into the 15% DRC of NR latex and stirred under a closed system for 24 h.

### Preparation of the hydrophobic CSSM

The SSM was cut into 3 × 3 cm pieces and then ultrasonically cleaned with acetone for 20 min, washed with DI water to remove the surface impurities, and dried at 50 °C for 2 h. Subsequently, the cleaned SSM was placed on the top of a crucible containing 0.5 g PDMS and the crucible was put into the muffle furnace for the vapor deposition^35^. The crucible was heated to 500 °C at 10 °C min^−1^ and then held at that temperature for 2 h. The liquid state PDMS was vaporized and decomposed to SiO_2_^35^, which was deposited on SSM. The obtained CSSM was referred to as “PDMS-coated mesh”. Finally, the PDMS-coated mesh was dipped in the in situ modified SiO_2_ generated in the NR latex by a sol–gel reaction and dried at 80 °C for 2 h. The SiO_2_ particles modified with OTES or HDTMS via the sol–gel reaction are represented as “MSi-O” and “MSi-H", respectively. The CSSM without PDMS is referred to as “NR/MSi-O” and “NR/MSi-H” for modified SiO_2_ by OTES and HDTMS generated in NR latex, respectively. The CSSMs are coded as “PDMS/NR/MSi-O” for the PDMS and modified SiO_2_ by OTES in the NR latex and as “PDMS/NR/MSi-H” for the PDMS and modified SiO_2_ by HDTMS in the NR latex.

### Characterization

The in situ modified SiO_2_ in NR latex was investigated using transmission electron microscopy (TEM; JEM-1400, JEOL, Japan) under an electron beam with an accelerating voltage of 80 kV. ^29^Si solid-state nuclear magnetic resonance spectroscopy (NMR) at 400 MHz (JEOL, JNM-ECZ-400R/S1, Japan) and Fourier transform infrared spectroscopy (FTIR; Thermo Fischer, Nicolet iS5, USA) with attenuated total reflection (ATR) mode over a wavenumber range of 4000 to 400 cm^−1^ with 32 scans at a 4 cm^−1^ resolution were used to confirm the in situ modified SiO_2_ generated in the NR matrix in terms of solid state without SSM. The surface morphology of the CSSMs was characterized using SEM (JSM-6610LV, JEOL, Japan) with an accelerating voltage of 15 kV. The static WCA was measured at ambient temperature by a contact angle goniometer (200-F1, Ramé-hart, USA) after applying a 6-μL distilled water droplet on the surface with a micro-syringe. For each sample, three water droplets were applied and measured.

### Mechanical durability

**Figure 1:**
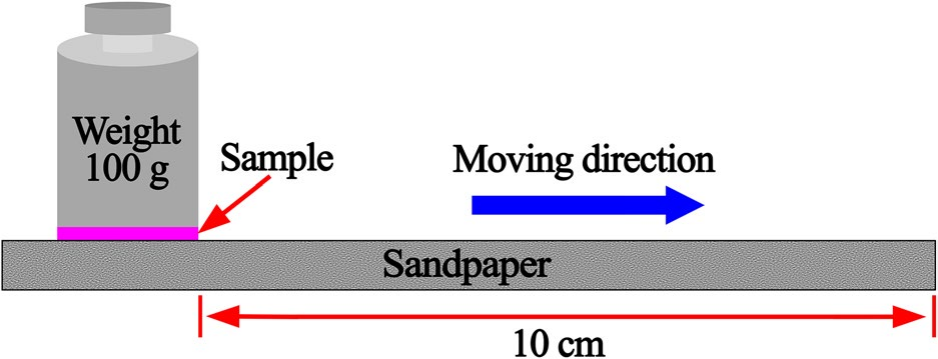
Illustration of the sandpaper-abrasion test.

A sandpaper abrasion test was performed on the hydrophobic coating of CSSM to characterize its mechanical durability. The hydrophobic CSSM was loaded with a weight of 100 g (with an area of 3.14 × 10^–4^ m^2^), and 400 grit sandpaper was used as an abrasion media, which was attached to the mesh bottom (Fig. 1), as reported previously^25^. The CSSM-attached weight was moved on the sandpaper for 10 cm, which was defined as one abrasion cycle. The WCA was measured after every abrasion cycle.

### Oil/seawater separation

**Figure 2:**
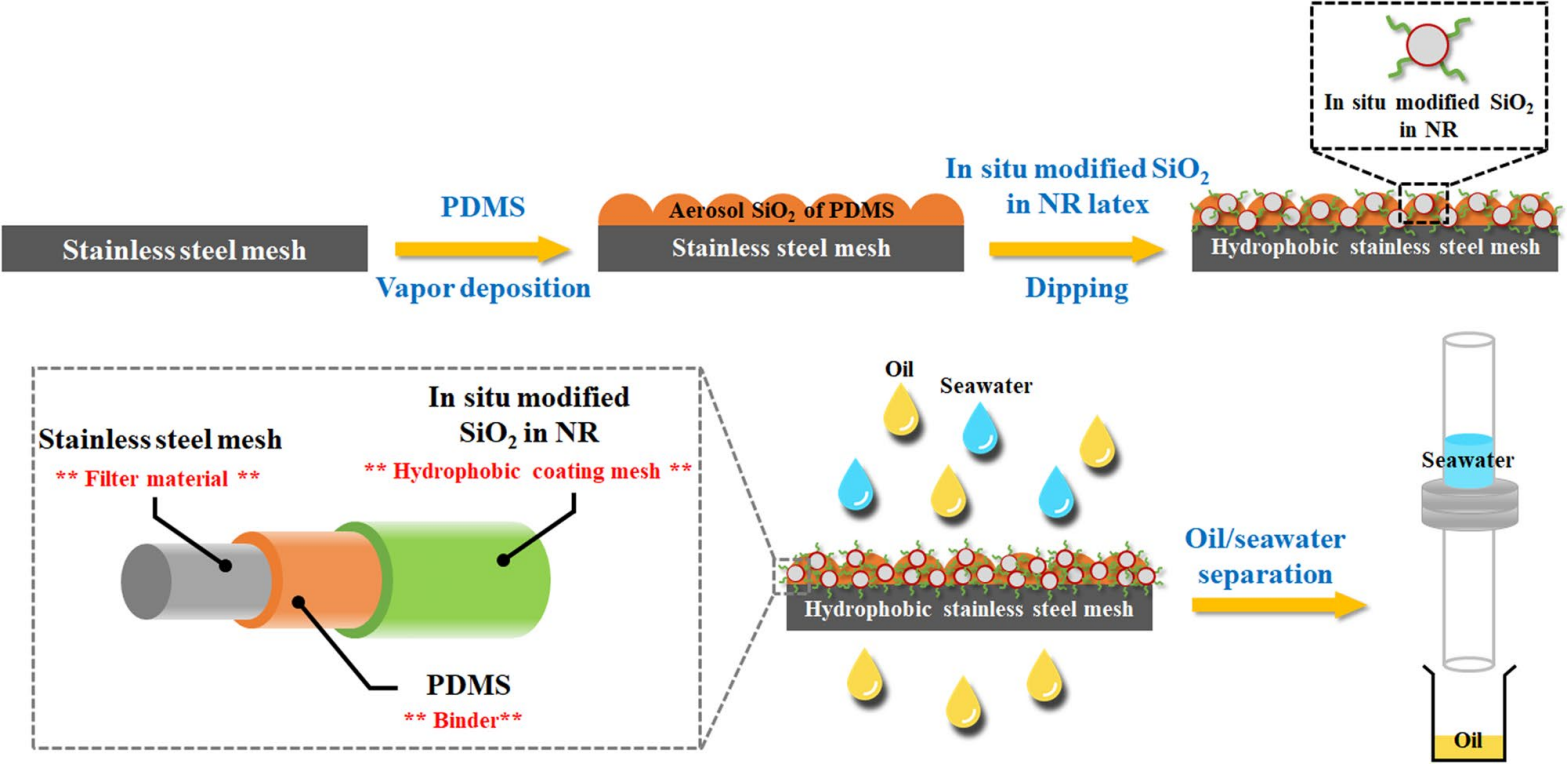
Overview of the experiment in this research.

In this study, the seawater was mixed with either chloroform [1:1 (v/v) ratio]or crude oil [9:1 (v/v) oil: seawater ratio] and their separation was investigated using the obtained CSSMs. The respective CSSM was held between two glass tubes. The separation device was placed vertically for separating chloroform (dyed red) from seawater, while it was tilted (ca. 45° from the vertical) for separating the crude oil/seawater (dyed blue) mixture. The respective mixture was poured onto the coated mesh and the oil or chloroform permeated through the test CSSM under gravity while the seawater was blocked and retained on the CSSM. The separation efficiency (*η*) and permeate flux were determined using Eqs. (1) and (2), respectively:

1 $$\upeta { = }\left( {\frac{{{\text{m}}_{{1}} - {\text{m}}_{{{\text{water}}}} }}{{{\text{m}}_{{0}} }}} \right) \times {100}$$

where *m*_0_ and *m*_1_ are the oil content (g) before and after separation, respectively, and *m*_water_ is the water content (g) in oil after separation.

2 $${\text{Permeate }}\;{\text{flux = }}\frac{{\text{V}}}{{{\text{S }} \times {\text{ t}}}}$$

where *V* is the volume of oil that permeates through the mesh (L), *S* is the effective area of the mesh (m^2^), and *t* is the permeating time (h). An overview of the experiment in this research is represented in Fig. 2.

## Results and discussion

**Characterization of the in situ modified SiO**_**2**_ **in NR latex**

**Figure 3:**
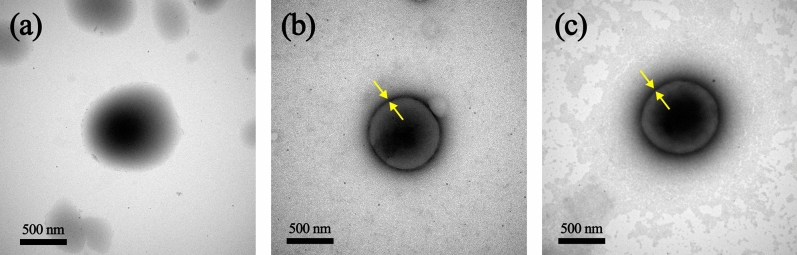
Representative TEM images of the (**a**) NR latex and (**b, c**) in situ modified SiO_2_ generated in NR latex with (**b**) OTES or (**c**) HDTMS.

Figure 3 shows the TEM images of the NR latex and in situ modified SiO_2_ generated in the NR latex. The NR particles in the NR latex had a diameter of about 1000 nm, while the in situ modified SiO_2_ by OTES and HDTMS generated in NR latex had an average particle size in the range of 720–770 nm with the network of SiO_2_ as a shell (yellow arrow) covered on the NR particles as a core. The hydrophobicity and steric effect of modified SiO_2_ networks cause the NR particle to shrink in size compared to the neat NR particle. The morphology of the in situ modified SiO_2_ generated in NR latex obtained in this study was similar to that in a previous work^36^.

**Figure 4:**
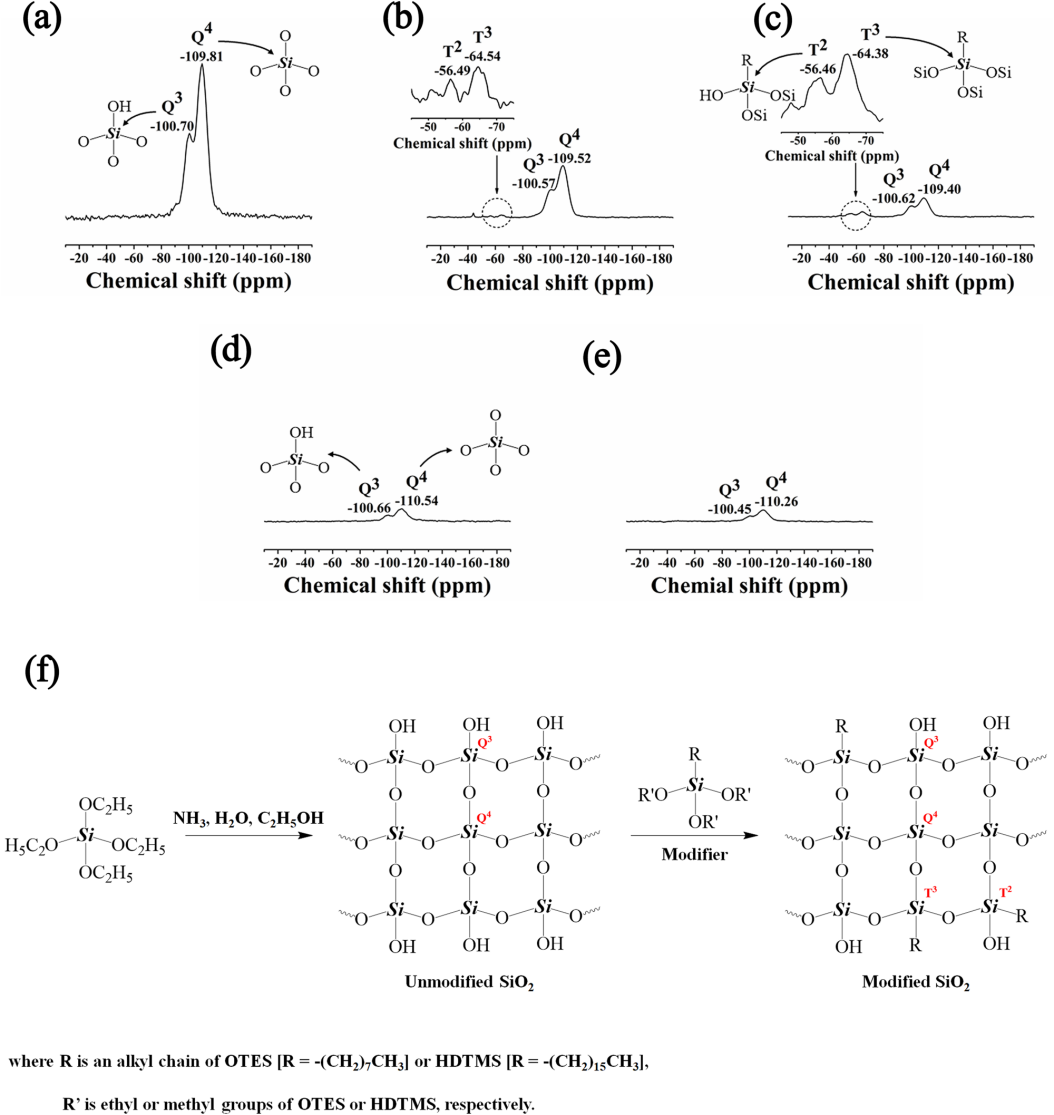
Representative ^29^Si solid-state NMR of the (**a**) unmodified SiO_2_, (**b**) MSi-O, (**c**) MSi-H, (**d**) NR/MSi-O, and (**e**) NR/MSi-H; and (**f**) the proposed mechanism of modified SiO_2_ via sol–gel reaction.

To confirm the successfully prepared in situ modified SiO_2_ generated in NR latex, ^29^Si solid-state NMR analysis was performed, with the results summarized in Fig. 4. The unmodified SiO_2_ spectrum showed peaks at − 109.81 ppm and − 100.70 ppm, which were attributed to Q^4^ and Q^3^, respectively. After modification, the intensities of the Q^4^ and Q^3^ peaks were significantly decreased and new signals appeared in the terms of the T^*n*^ group at − 64.54 and − 56.49 (Fig. 4b) ppm for the OTES-modified SiO_2_ and at − 64.38 and − 54.46 (Fig. 4c) ppm for the HDTMS-modified SiO_2_, which corresponded to T^3^ and T^2^, respectively. These clearly confirm that the condensation reaction between the alkyl silanes and the silanol groups on the SiO_2_ particles had occurred^37,38^ (Fig. 4f). It is interesting to note that after generating in situ modified SiO_2_ in NR latex, the T^3^ and T^2^ peaks disappeared and the intensity of the Q^4^ and Q^3^ peaks were significantly decreased. This may be due to the interference from the NR covering the SiO_2_ networks.

**Figure 5:**
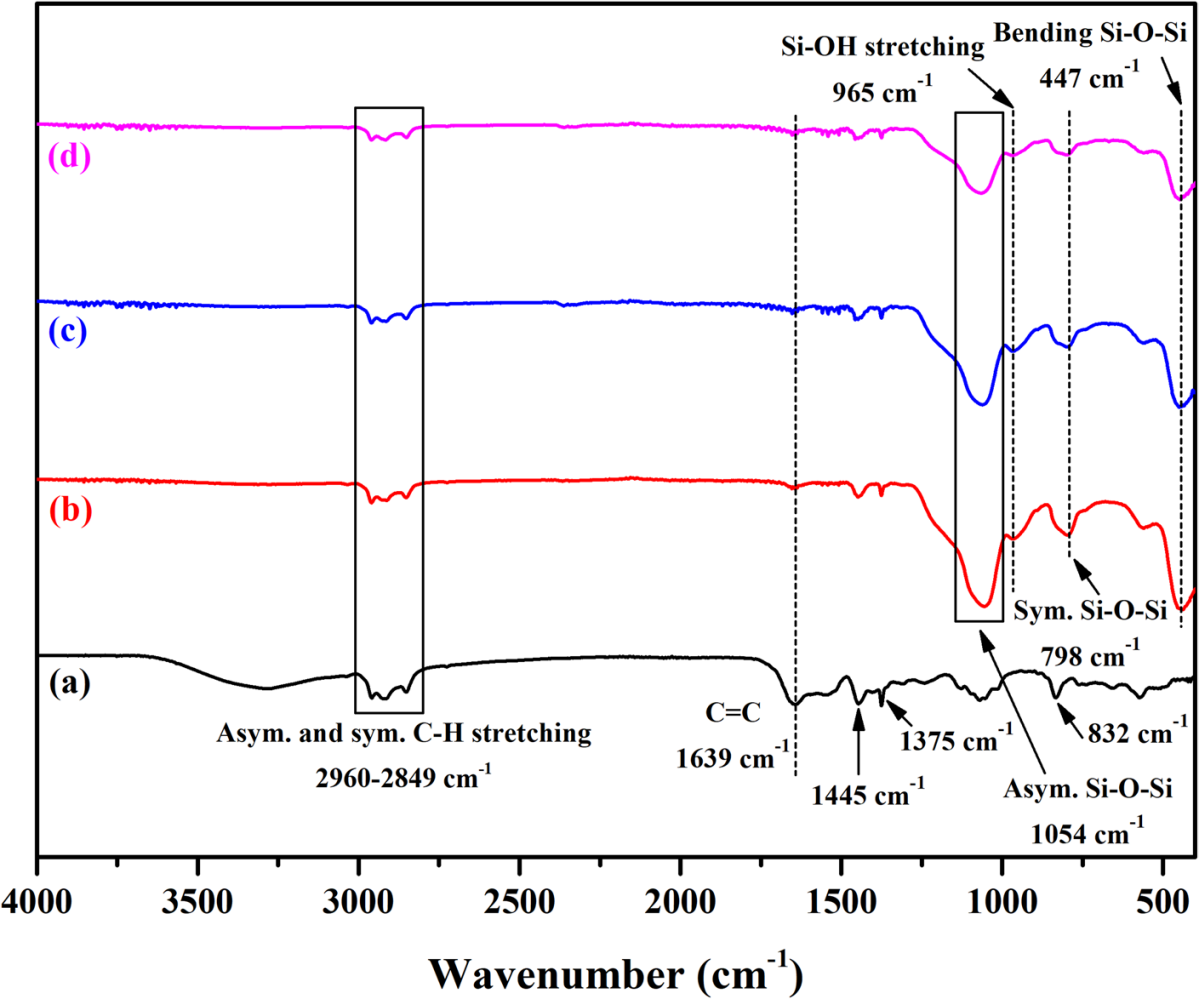
Representative FTIR spectra of (**a**) neat NR, (**b**) in situ unmodified SiO_2_ generated in the NR latex, and (**c**) OTES- and (**d**) HDTMS-modified SiO_2_ generated in the NR latex.

In addition, the FTIR spectra of the neat NR, in situ SiO_2_ generated in the NR latex, and the in situ modified SiO_2_ by OTES and HDTMS generated in the NR latex are shown in Fig. 5. The neat NR exhibited the asymmetric and symmetric stretching of C–H groups at 2960–2849 cm^−1^, C=C at 1639 cm^−1^, –CH_2_– symmetric stretching and –CH_3_ asymmetric stretching at 1445 cm^−1^, asymmetric –CH_3_ bending at 1375 cm^−1^, and C–H out-of-plane bending at 832 cm^−1^ of the *cis*-1,4-polyisoprene^39–41^. In the presence of the in situ formed SiO_2_ in the NR latex, new absorption peaks were observed at 1054, 798, and 447 cm^−1^ corresponding to the asymmetric stretching, symmetric stretching, and bending vibration of the Si–O–Si, respectively^42,43^. The band at 965 cm^−1^ was due to the stretching vibration of silanol (Si–OH) groups^42,43^. After SiO_2_ modification by OTES or HDTMS, the peak intensity for the absorption peaks of Si–O–Si and Si–OH was decreased compared to the unmodified ones. These results support that the in situ modified SiO_2_ particles had the –OH groups replaced by the long chain alkyl groups of OTES or HDTMS.

The static WCA of the SiO_2_ NPs before and after modification was also examined in order to confirm the sol–gel reaction of OTES and HDTMS. The results revealed that the static WCA of the SiO_2_ NPs before and after modification was significantly changed as follows: 118.3° (SiO_2_: Si), 134.8° (MSi-O) and 147.2° (MSi-H). Additionally, the NR/Si (in situ unmodified SiO_2_ in NR latex) CSSM showed the hydrophilic mesh with a static WCA of 54.3° compared to the NR/MSi-O (129.1°) and NR/MSi-H (134.0°) CSSMs [see in supplementary information (SI)]. These results confirmed that the in situ SiO_2_ was successfully modified on the surface of the NR latex by the long chain alkyl groups of OTES or HDTMS via a sol–gel reaction.

### Characterization of the CSSMs

**Figure 6:**
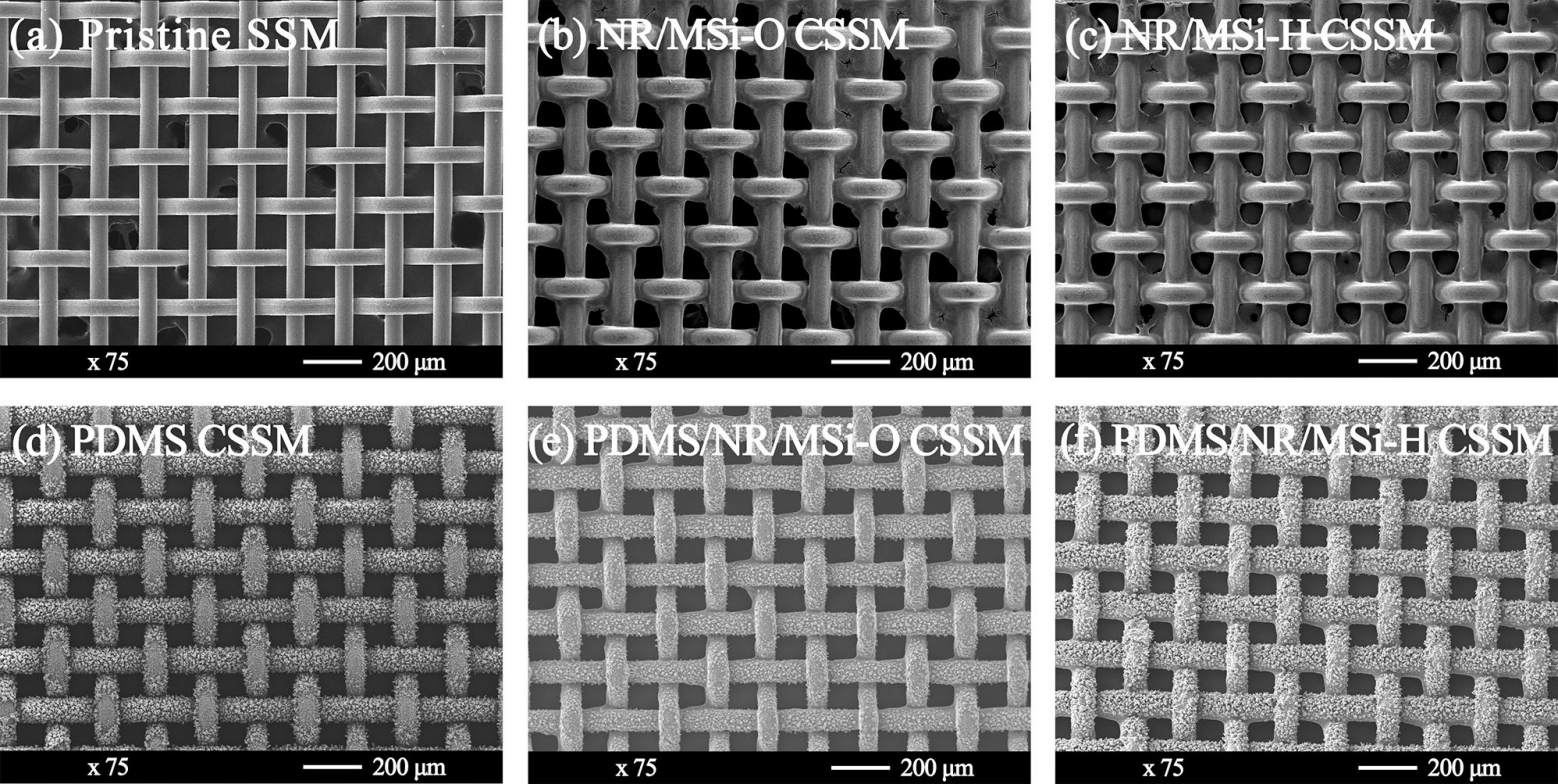
Representative SEM images of the (**a**) pristine SSM, and the (**b**) NR/MSi-O, (**c**) NR/MSi-H, (**d**) PDMS, (**e**) PDMS/NR/MSi-O, and (**f**) PDMS/NR/MSi-H CSSMs.

The SEM images (Fig. 6) revealed the surface morphology of the SSM and the CSSMs after coating by PDMS and the in situ modified SiO_2_ generated in the NR latex. The surface of the pristine SSM was smooth and the pristine SSM was woven by a single layer of metal wires with an average diameter of 60 μm to form a square pore of ~ 106 μm. After coating, the average pore size of the NR/MSi-O and NR/MSi-H CSSMs tended to be decreased (Fig. 6b,c). The PDMS-CSSM (Fig. 6d) showed aggregates or agglomerates of aerosol SiO_2_ particles from the vapor deposition of PDMS^35^. The CSSM surface became rougher and the pore size of PDMS-CSSM was slightly decreased compared to the uncoated SSM. After coating with the in situ modified SiO_2_ generated in the NR latex, the NR latex covered the PDMS (Fig. 6e,f). In addition, both the aerosol SiO_2_ particles from PDMS and the in situ modified SiO_2_ were fused into large aggregates or agglomerates.

**Figure 7:**
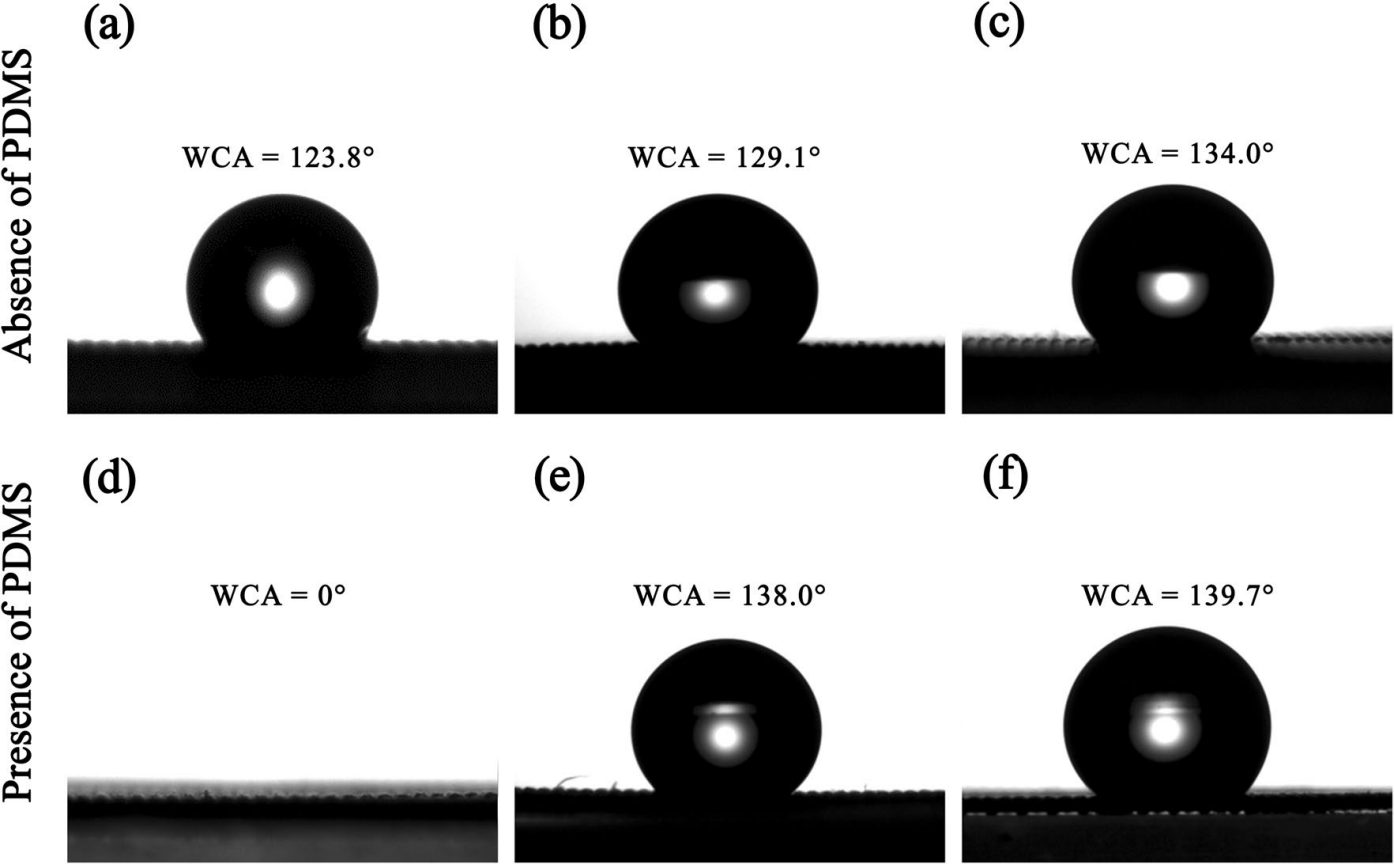
The static WCA of the (**a**) pristine SSM, and the (**b**) NR/MSi-O, (**c**) NR/MSi-H, (**d**) PDMS, (**e**) PDMS/NR/MSi-O, (**f**) and PDMS/NR/MSi-H CSSMs.

Comparison of the static WCA in the absence and presence of PDMS is shown in Fig. 7. In the absence of PDMS, the pristine (uncoated) SSM had a static WCA of 123.8°, demonstrating its hydrophobic nature. After coating with the in situ modified SiO_2_ generated in the NR latex, the static WCA of the CSSMs were increased to 129.1° for NR/MSi-O and 134.0° for NR/MSi-H. In the presence of PDMS, the static WCA of hydrophilic PDMS-CSSM became 0°due to the aerosol SiO_2_ particles that were formed after vapor deposition at 500 ^o^C^35^. It is interesting to note that the hydrophobicity of PDMS/NR/MSi-O and PDMS/NR/MSi-H CSSMs significantly increased with a static WCA of 138.0° and 139. 7°, respectively. This means that the enhanced surface roughness induced by PDMS could increase the adhesion between the aerosol SiO_2_ particles from PDMS and the in situ modified SiO_2_, resulting in the increased hydrophobicity of the CSSMs. Accordingly, hydrophobic CSSMs (PDMS/NR/MSi-O and PDMS/NR/MSi-H) were successfully prepared by coating with PDMS and in situ modified SiO_2_ generated in the NR latex. Thus, not only the surface roughness of SSM but also the hydrophobic modified SiO_2_ NPs were the important factors to increase/decrease the hydrophobicity of CSSM.

### Mechanical durability of hydrophobic CSSMs

**Figure 8:**
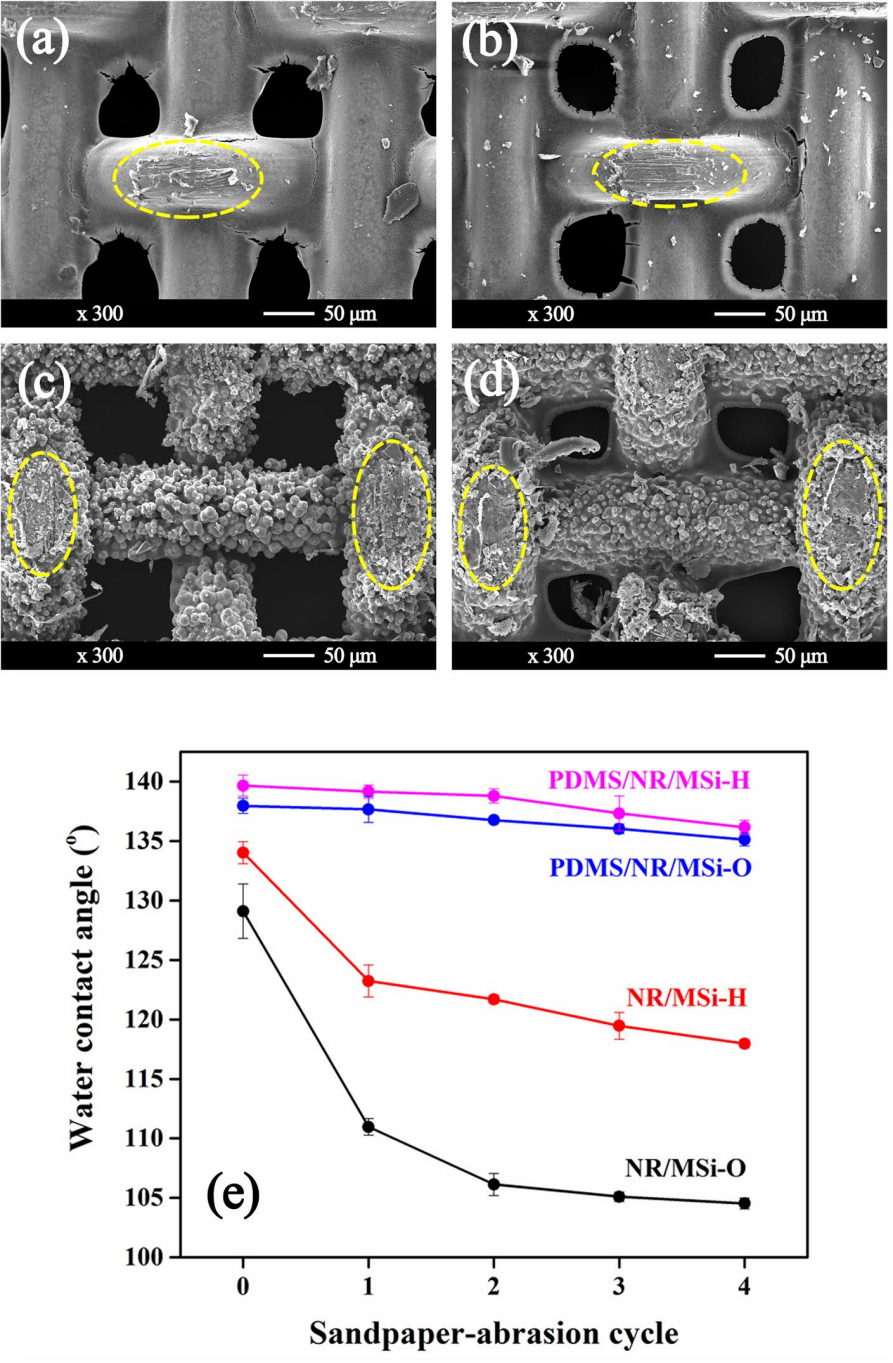
(**a–d**) Representative SEM images showing the surface morphologies after the fourth sandpaper-abrasion cycle of (**a**) NR/MSi-O, (**b**) NR/MSi-H, (**c**) PDMS/NR/MSi-O, and (**d**) PDMS/NR/MSi-H, (**e**) The static WCA of the hydrophobic CSSMs after the sandpaper-abrasion test.

Mechanical durability is an important required trait for the practical application of any hydrophobic CSSM. Therefore, a sandpaper-abrasion test was used in this study to test the robustness of the prepared SSM and CSSMs. The morphology of the SSM and CSSM samples after four cycles of the sandpaper-abrasion test was observed from the SEM images (Fig. 8a-d), where the surface was clearly damaged along the scratched direction in all cases (yellow dashed frames in Fig. 8). However, the weight loss was too low to reliably estimate. Accordingly, the relationship between the static WCA and the sandpaper-abrasion cycles was examined. The static WCA values of the CSSMs without PDMS were clearly decreased from the first cycle sandpaper-abrasion testing (129.1°–104.5° for NR/MSi-O and 134.0°–118.0° for NR/MSi-H), revealing a reduced hydrophobicity. The static WCA values for the CSSMs with PDMS were higher than those without, where the static WCA values of PDMS/NR/MSi-O and PDMS/NR/MSi-H seemed to remain almost constant after four cycles of the sandpaper-abrasion test (Fig. 8e). This result confirmed that PDMS acted as a binder to enhance the hydrophobicity of the CSSM and improve its mechanical durability. Therefore, the PDMS/NR/MSi-O and PDMS/NR/MSi-H CSSMs were selected for oil/seawater and chloroform/seawater separation to evaluate their potential for practical application.

### Separation of oil or chloroform from seawater

**Figure 9:**
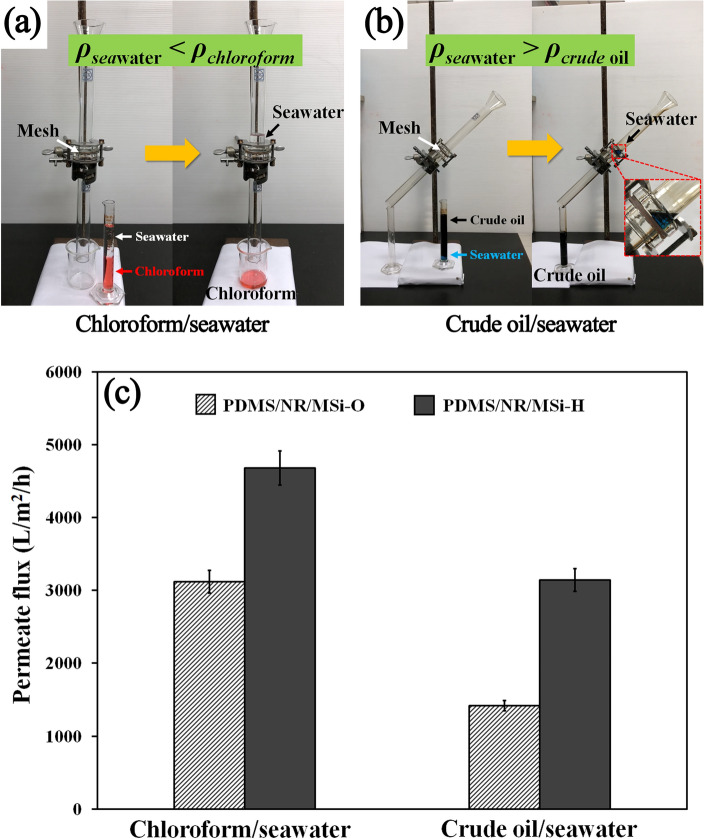
(**a, b**) Schematic diagram showing the separation process of (**a**) chloroform/seawater and (**b**) crude oil/seawater. (**c**) The permeate fluxes through the CSSMs of chloroform/seawater and crude oil/seawater.

The separation of chloroform or crude oil from seawater was examined using the two hydrophobic CSSMs (PDMS/NR/MSi-O and PDMS/NR/MSi-H). The criteria for choosing the non-aqueous (oil) phase for separation was based upon density and immiscibility compared to seawater, where the density of chloroform (1.489 g/cm^3^) and crude oil (0.817 g/cm^3^) is higher and lower, respectively, than that of seawater (1.017 g/cm^3^). Therefore, the chloroform and crude oil easily phase separated below and above, respectively, the seawater phase. The experimental setup for the separation process is shown schematically in Fig. 9a,b. The test hydrophobic CSSM was held between two glass tubes and the separation device was placed vertically for chloroform (red solution)/seawater, while it was tilted (45° from the vertical) for separating the crude oil/seawater (blue solution). The respective mixture was poured onto the test CSSM and the chloroform or crude oil permeated through the CSSM under gravity, while the seawater was blocked on the mesh. The permeate flux of the hydrophobic CSSM in each condition is shown in Fig. 9c, which revealed that the permeate fluxes in the case of chloroform/seawater were higher than that of the crude oil/seawater mixture, due to their different viscosities. The viscosity of crude oil (99.6 cP)^44^ is higher than that of chloroform (0.514 cP)^45^, leading to the lower permeate flux and higher separation time. Moreover, the permeate flux with the PDMS/NR/MSi-H CSSM was significantly higher than that with the PDMS/NR/MSi-O due to their different hydrophobicity.

**Figure 10:**
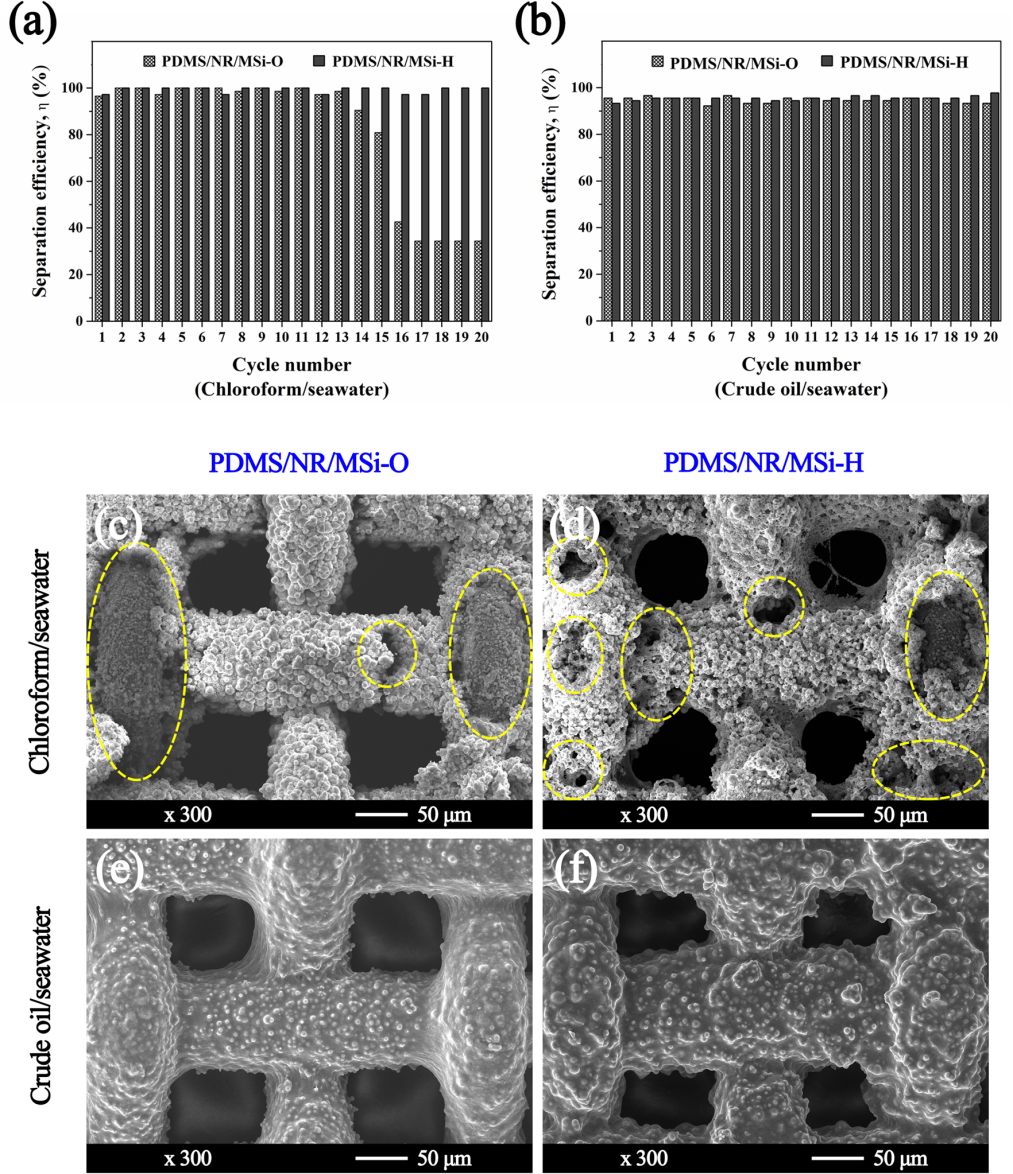
The (**a, b**) separation efficiencies of (**a**) chloroform/seawater and (**b**) crude oil/seawater, and (**c–f**) representative SEM images showing the surface morphologies after 20 separation cycles of (**c, d**) chloroform/seawater and (**e, f**) crude oil/seawater through (**c, e**) PDMS/NR/MSi-O and (**d, f**) PDMS/NR/MSi-H.

The separation efficiency of the two hydrophobic CSSMs (PDMS/NR/MSi-O and PDMS/NR/MSi-H) is shown in Fig. 10a,b for 20 separation cycles. The separation efficiencies were up to 81.9% (PDMS/NR/MSi-O) and 99.3% (PDMS/NR/MSi-H) for the chloroform/seawater mixture. More importantly, after 13 cycles of separation, the separation efficiency was gradually reduced by about 10 and 20% after the 14th and 15th cycle, respectively, and significantly reduced to ~ 40% after the 16th cycle for PDMS/NR/MSi-O. Compared with the crude oil/seawater mixture, the separation efficiencies of both PDMS/NR/MSi-O and PDMS/NR/MSi-H were comparable at approximately 95–96% after 20 separation cycles.

The surface morphology of the two hydrophobic CSSMs (PDMS/NR/MSi-O and PDMS/NR/MSi-H) after the 20th separation cycle was examined from the SEM images (Fig. 10c–f). In the chloroform/seawater mixture, the NR was dissolved in the chloroform (yellow dashed frames in Fig. 10) making the CSSM surface easily damaged during the separation process. In contrast, the surface was only swelled and not damaged with the crude oil/seawater mixture. In conclusion, this material can be applied as a filter material to separate a crude oil/seawater mixture.

Finally, comparison of the WCA, separation efficiency, and reusability of several materials is summarized in Table 1. Although, the WCA of the optimal hydrophobic CSSM (PDMS/NR/MSi-H) in the present work was lower than that of others, the PDMS/NR/MSi-H CSSM exhibited a high separation efficiency and reusability. It is important to note that the separation efficiency of each material depends on several factors, such as the hydrophobicity of material or the oil properties.

**Table 1:** Comparison of oil/water separation efficiencies of various materials.

Substrate	WCA (°)	Non-aqueous/water mixture	Separation efficiency (%)	Permeate flux (L/m^2^/h)	Cycle number	Ref
PDMS/NR/MSi-H CSSM	139.7	Chloroform/seawater	99.3	4677	20	This study
		Crude oil/seawater	> 95	3143	20	This study
TCMS^a^ copper mesh	159	n-Hexane, toluene, chloroform, and dichlromethane/water	99.9	> 2.2 × 10^5^	80	^15^
Cu-PDA^b^/SH^c^ copper mesh	152.4	Silicon oil/distilled water	> 90	4507	–	^46^
VTMO^d^/TS720^e^ CSSM	152 ± 0.8	Petroleum ether/water	92.58	–	50	^1^
Lauric acid modified copper mesh	155.5 ± 3	Petroleum, hexane, toluene, gasoline, and diesel oil/water	> 93	–	10	^47^
ODT^f^ modified SSM	145	Gasoline, diesel, engine oil, hexane, and paraffin oil/water	> 99.0	–	10	^48^
PDMS CSSM	> 150	Chloroform/water	98.9	1.5 × 10^5^	60	^35^

^a^Trichloromethylsilane.

^b^Polydopamine.

^c^1-Dodecanethiol.

^d^Vinyltrimethoxysilane.

^e^Hydrophobic fumed silica.

^f^*n*-Octadecylthiol.

## Conclusion

In summary, this work successfully fabricated hydrophobic CSSMs for oil/water separation. The ^29^Si solid-state NMR and FTIR analyses confirmed that the in situ modified SiO_2_ with OTES or HDTMS generated in the NR latex was successfully prepared via the sol–gel technique. The hydrophobicity of the SSM was improved by the in situ modified SiO_2_ in the NR latex, which exhibited a WCA of 138.0° for PDMS/NR/MSi-O and 139.7° for PDMS/NR/MSi-H. Moreover, the presence of SiO_2_ particles from PDMS enhanced the roughness and the mechanical durability. Both PDMS/NR/MSi-O and PDMS/NR/MSi-H were more selective for crude oil than chloroform since the NR could be easily dissolved in chloroform during the separation process. The PDMS/NR/MSi-H showed a better permeate flux and separation efficiency with both the chloroform/seawater (99.3%) and crude oil/seawater (95.56%) mixtures than PDMS/NR/MSi-O, and it could be reused for at least 20 cycles. Accordingly, the hydrophobic PDMS/NR/MSi-H CSSM obtained in this study could be applied for removal of oil spills.

### Supplementary Information

Supplementary Information.

## Data Availability

The dataset generated and/or analyzed during the current study are available from the corresponding authors on reasonable request.
