# Supplementary material for: Modified silica-based double-layered hydrophobic-coated stainless steel mesh and its application for oil/seawater separation
Source: Sci Rep. 2024 Jan 6;14:731. doi: 10.1038/s41598-024-51264-8 (PMC10771502; doi:10.1038/s41598-024-51264-8)
Supplement: Supplementary file 1 — Supplementary Information. [file 41598_2024_51264_MOESM1_ESM.pdf]

Modified silica-based double-layered hydrophobic-coated stainless steel mesh and its application for oil/seawater separation

Aunchalee Deachophon, Thiti Bovornratanaraks, Sirilux Poompradub

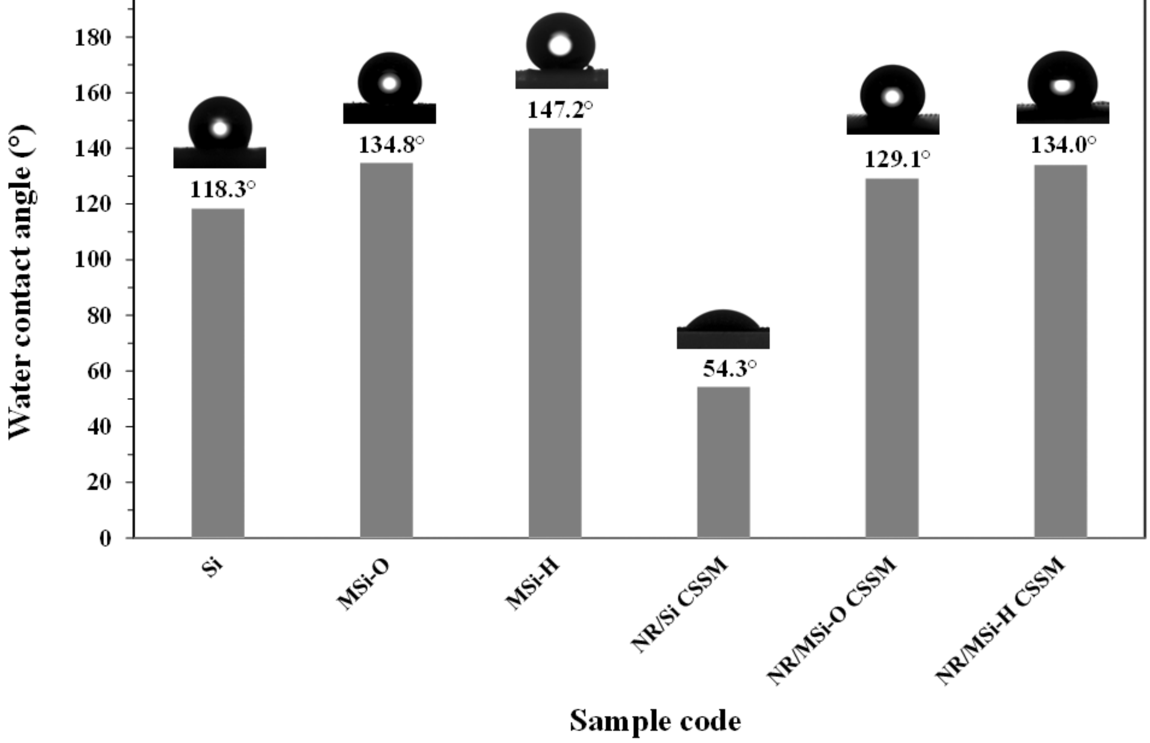

Supplementary Figure. The static WCA of the SiO_2 NPs before/after modification and CSSMs with NR/Si (in situ unmodified SiO_2 in NR latex), NR/MSi-O and NR/MSi-H, respectively.
